# Allele-specific and multiplex PCR based tools for cost-effective and comprehensive genetic testing in Congenital Adrenal Hyperplasia

**DOI:** 10.1016/j.mex.2022.101748

**Published:** 2022-05-31

**Authors:** Lavanya Ravichandran, Deny Varghese, Parthiban R, Asha H. S, Sophy Korula, Nihal Thomas, Aaron Chapla

**Affiliations:** a Department of Endocrinology, Diabetes and Metabolism, Christian Medical College, Ida Scudder Road, Vellore, Tamil Nadu, India; b Department of Paediatric Endocrinology, Christian Medical College, Ida Scudder Road, Vellore, Tamil Nadu, India; c Regional Centre for Biotechnology, Faridabad, India

**Keywords:** 21 – hydroxylase deficiency, Congenital Adrenal Hyperplasia, Allele Specific PCR, Multiplex PCR

## Abstract

Congenital Adrenal Hyperplasia (CAH) is an autosomal recessive disorder due to enzyme defects in adrenal steroidogenesis. Several genes code for these enzymes, out of which mutations in the *CYP21A2* gene resulting in 21 hydroxylase deficiency, contribute to the most common form of CAH. However, pseudogene imposed challenges complicate genotyping *CYP21A2* gene, and there is also a lack of comprehensive molecular investigations in other genetic forms of CAH in India. Here, we describe a cost-effective, highly specific, and sensitive Allele Specific PCR (ASPCR) assay designed and optimized in-house to screen eight common pathogenic mutations in the *CYP21A2* gene. We have also established and utilized a multiplex PCR assay for target enrichment and Next-generation sequencing (NGS) of *CYP11B1, CYP17A1, POR,* and *CYP19A1* genes. Following preliminary amplification of the functional gene *CYP21A2*, ASPCR based genotyping of eight common mutations - P30L, I2G, 8BPdel, I172N, E6CLUS (I235N, V236E, M238K) V281L, Q318X, and R356W was carried out. These results were further validated using Sanger and Next-generation sequencing. Once optimized to be specific and sensitive, the advantage of ASPCR in *CYP21A2* genotyping extends to provide genetic screening for both adult and paediatric subjects and carrier testing at a low cost and less time. Furthermore, multiplex PCR coupled NGS has shown to be cost-effective and robust for parallel multigene sequencing in CAH.

## Graphical abstract

**Figure:**
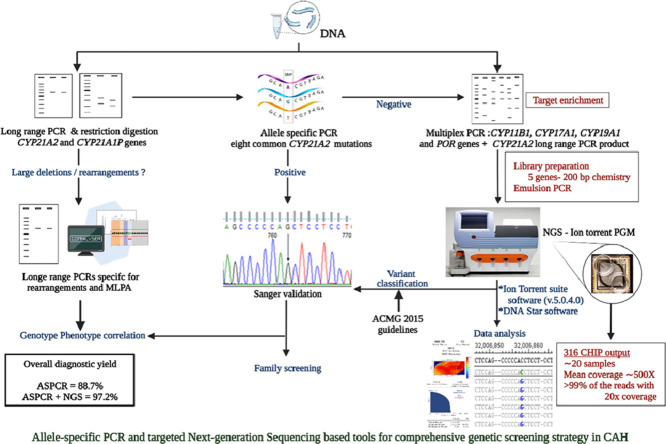

**Specifications table**

**Table:** 

Subject Area;	Biochemistry, Genetics and Molecular Biology
More specific subject area;	*Genotyping with Allele Specific PCR and target enrichment with multiplex PCR*
Protocol name;	*Allele Specific and Multiplex PCR for genetic testing in CAH*
Reagents/tools;	*Gentra Puregene DNA extraction kit from QIAGEN®* *TaKaRa LA PCR^TM^ Kit (ver.2.1)* *EmeraldAmp® Max PCR master mix* *QIAGEN® Multiplex PCR Kit*
Experimental design;	*1. Long-range PCR for CYP21A2 gene amplification followed by Allele Specific PCR for screening eight hotspot mutations*. *2.Multiplex PCR for target enrichment of CYP11B1, CYP17A1, CYP19A1and POR genes*
Trial registration;	*N/A*
Ethics;	*N/A*
Value of the Protocol;	• *ASPCR - highly specific and sensitive to identify eight pseudogene derived mutations in the CYP21A2 gene.* • *Multiplex PCR – cost-effective and robust for target enrichment of CAH related genes* • *Together aids in comprehensive genetic screening for CAH in clinical settings*

## Description of the protocol

### DNA extraction and long-range PCR

**Fig. 1:**
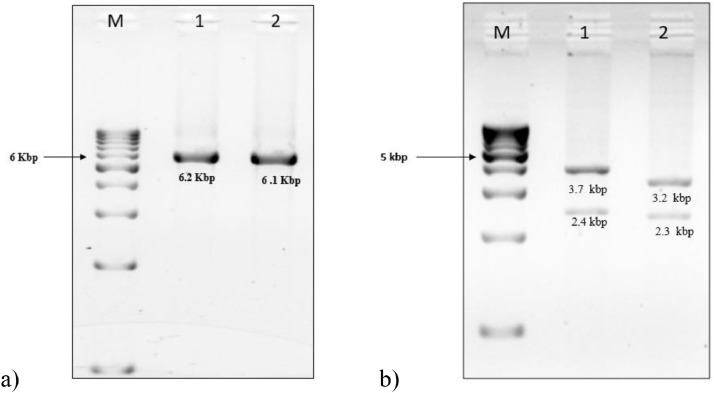
a) 1% Agarose gel image of locus-specific amplification of *CYP21A2* and *CYP21A1P* genes. M-1 Kbp ladder, lane 1: functional gene product *CYP21A2* (6.2 kbp) amplified with primers CYP779f/Tena36F and lane 2: pseudogene product *CYP21A1P* (6.1 kbp) amplified with primers CYP779f/ XA-36F adapted from *Lee et al*[1]. b) Agarose gel image (1%) - restriction digestion of *CYP21A2* and *CYP21A1P* genes with TaqI. The product sizes of digested products from the functional gene were 3.7 kbp & 2.4 kbp shown in lane 1 and the digested products from the pseudogene were 3.2 kbp & 2.3 kbp shown in lane 2.

**Fig. 2:**
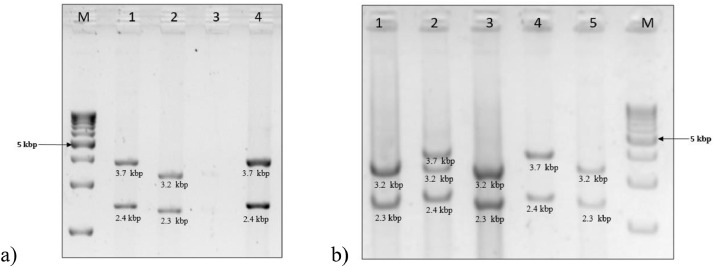
a) Restriction digestion [1] results with TaqI in large gene conversion on 1% agarose gel electrophoresis – Lane 1 and 2 shows normal restriction digested fragments of functional and pseudogene amplified with long range PCR in the negative control. In one subject, there was no amplification with functional gene primers CYP779f/Tena36F2, and so there were no digested products as seen in lane 3. However, there was amplification with the pseudogene primers (CYP779f/ XA-36F) with a restriction digestion pattern similar to the functional gene, as shown in lane 4. This suggests a homozygous large gene conversion involving the proximal end of *CYP21A2* and the distal end of *CYP21A1P* genes. b) Restriction digestion results in large 30 kbp deletion on 1% agarose gel electrophoresis: Lane 4 and 5 show normal restriction digested fragments of the functional and pseudogene in the negative control. Lane 1 shows a restriction digestion pattern of a sample with homozygous 30 kbp deletion. Since the deletion involves forming a chimeric (fusion) gene with the proximal end of *CYP21A1P* and the distal end of *CYP21A2* genes, there is no amplification with pseudogene primers (CYP779f/ XA-36F). However, the product amplified with functional gene primers (CYP779f/Tena36F2) gives a restriction digestion pattern similar to that of the pseudogene. A heterozygous 30 kbp deletion on one allele results in amplification with both the primer sets, but the product from functional gene primers produces three restriction digestion bands resulting in a combination of functional and pseudogene, as seen in lane 2.

DNA extraction was carried out with 2 ml EDTA whole blood using Gentra Puregene kit from QIAGEN® (Hilden, Germany) and quantified using NanoDrop™ spectrophotometer. Long-range PCRs were utilized for locus-specific amplification of the functional gene *CYP21A2* (6.2 kbp) and pseudogene *CYP21A1P* (6.1 kbp) with TaKaRa LA PCR^TM^ Kit (ver.2.1) using previously published protocols [1]. In addition, the results were validated with TaqI restriction digestion [1]. Based on these results, samples suspected for large 30 kbp deletion and large gene conversion were validated with MLPA and additional long-range PCRs with specific primers for these rearrangements described previously by *Greene et al* [2]. Figs. 1 and 2 show results of locus-specific amplification and restriction digestion of the above genes and their interpretation in identifying rearrangements

### Allele Specific PCR (ASPCR) for screening eight hotspot mutations in *CYP21A2* gene

The long-range PCR product of the *CYP21A2* gene was utilized as a template for Allele Specific PCR (ASPCR) to genotype eight common hotspot mutations in the *CYP21A2* gene. ASPCR, a modified application of conventional PCR technique, is a strategy to detect point mutations and small deletions by deliberately introducing mismatches in the primers. Primer designing is crucial in ASPCR to generate detectable amplicons from the mutation target while minimizing false priming at the non-target allele. A wild-type (WT) primer complementary to the normal sequence is designed for each target sequence harboring the hotspot mutation. A mutant (MT) primer complementary to the 3’ terminal base of the mutation under study is also designed for the same target. A reverse primer common for both the WT and MT is designed to maintain the same size for both WT and MT products. The wild type primer will provide amplification only with the wild type allele and there is no amplification when the allele is mutated.

Similarly, a mutant primer can amplify only the DNA sequence that carries the mutation. This enables the identification of the hotspot mutation under simple PCR conditions. Mismatches at the penultimate bases are often intentionally added to increase the specificity of the ASPCR [3]. If the terminal destabilization is weak, a strong destabilizing mismatch is added at the penultimate base and vice versa with a strong destabilization at the terminal base. Two WT forward primers were designed for I2G splice site mutation, including two WT alleles A and C. For all the eight hotspot mutations, common internal control primers were designed in such a way, it is amplified with both WT and MT alleles.

#### *Pre-clean up*

The long-range PCR product of the *CYP21A2* gene is purified using Agencourt AMPure XP (Beckman Coulter Life Sciences, USA) magnetic beads with the following protocol. This cleaned up product is used as a template for ASPCR



### Standardization of ASPCR conditions

The ASPCR was in house standardized with Emerald Amp® Max PCR master mix (Takara Bio Inc, Japan) in 15 µl reaction volume. The primer sequences are given below in table 1.

• WF- Wildtype Forward, MF- Mutant Forward, F-Forward, R-Reverse, WR-Wildtype Reverse, MR-Mutant Reverse.
• *The underlined sequences were adapted from *Lee et al*. [1]

**Table 1:** Primer sequences for in house designed ASPCR to genotype eight common pseudogene derived mutations in *CYP21A2* gene.

S.NO	PRIMER NAME	5' PRIMER SEQUENCE 3'
1	CAH ARMS INTERNAL CONTROL F	TGTGGCGGTGTAGTTGGTGTGG
2	CAH ARMS INTERNAL CONTROL R	GGGGACTTGTTCAGGGTGGGGA
3	CAH P30L WF	CTCCGGAGCCTCCACCTCCC
4	CAH P30L MF	CTCCGGAGCCTCCACCTCCT
5	CAH P30L R	TCAGTTCAGGACAAGGAGAGGCT
6	CAH I2G WF [C allele]	TTCCCACCCTCCAGCCCCCGC
7	CAH I2G WF [A allele]	TTCCCACCCTCCAGCCCCCTA
8	CAH I2G MF [G allele]	TTCCCACCCTCCAGCCCCCGG
9	CAH I2G R	TCAGTTCAGGACAAGGAGAGGCT
10	CAH 8BPDEL WF	CCGGACCTGTCCTTGGGAGACTAC
11	CAH 8BPDEL MF	TACCCGGACCTGTCCTTGGTC
12	CAH 8BPDEL R	*ATGCAAAAGAACCCGCCTCATAG
13	CAH I172N WF	CTCTCCTCACCTGCAGCATCAT
14	CAH I172N MF	CTCTCCTCACCTGCAGCATCAA
15	CAH I172N R	GAGGGTGTTTGCTGTGGTCTCA
16	CAH EX 6 CLUS WF	ATCACATCGTGGAGATGCAGCT
17	CAH EX 6 CLUS MF	GAGGGATCACAACGAGGAGAA
18	CAH E6 CLUS R	*AGCCCCAGCCGCACAGTGCTCA
19	CAH V281L WF	GACAGCTCCTGGAAGGGCACG
20	CAH V281L MF	GACAGCTCCTGGAAGGGCACT
21	CAH V281L R	TCTCCCAGACCGTCTCATCCA
22	CAH Q318X WF	CCAGATTCAGCAGCGACTGC
23	CAH Q318X MF	CCAGATTCAGCAGCGACTGT
24	CAH Q318X R	CTCGCACCCCAGTATGACT
25	CAH R356W F	*CTGGAGCCACTGGTCCATCCAA
26	CAH R356W WR	GCTAAGAGCACAACGGGCCG
27	CAH R356W MR	GCTAAGAGCACAACGGGCCA

Optimal annealing temperature and template concentration were utilized with appropriate positive and negative controls, and the below conditions were finalized to achieve optimal results. P30L hotspot mutation required primer redesigning to overcome false-positive results. Change in DNA extraction techniques can also affect the specificity of ASPCR and might require further standardization of the template concentration used. Details of the ASPCR reaction mix and program are mentioned in Tables 2a and 2b

**Table 2a:** ASPCR reaction Mix.

Contents	Volume
EmeraldAmp® Max PCR master mix Forward primer Reverse primer Internal forward primer Internal reverse primer Template* Sterile water	7.5 µl 1 µl 1 µl 1 µl 1 µl 1 µl 2.5 µl
Total	15 µl

*Template - cleaned up PCR product of *CYP21A2* gene (diluted concentration: 5-8 ng/µl).

Primer concentration used: 10 pmol/µl.

**Table 2b:** ASPCR - Thermal cycler conditions.

Stage 1×1	Initial denaturation	95 °C	5 minutes
Stage 2 × 20	Denaturation	98 °C	10 seconds
	Annealing	68 °C 70 °C (for I2G only)	30 seconds
	Extension	72 °C	1 minute
Stage 3 × 1	Final extension	72 °C	5 minutes

**Fig. 3:**
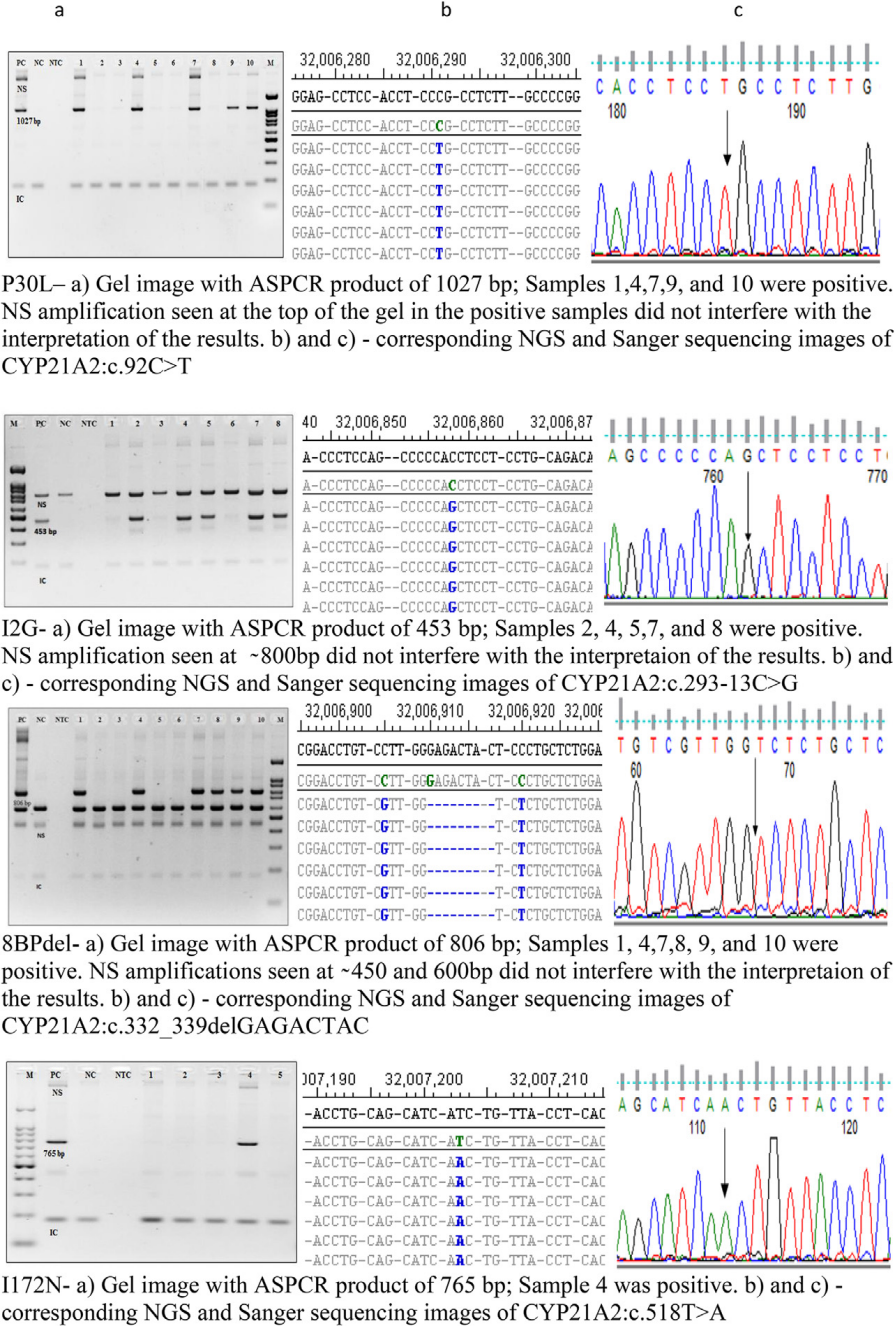

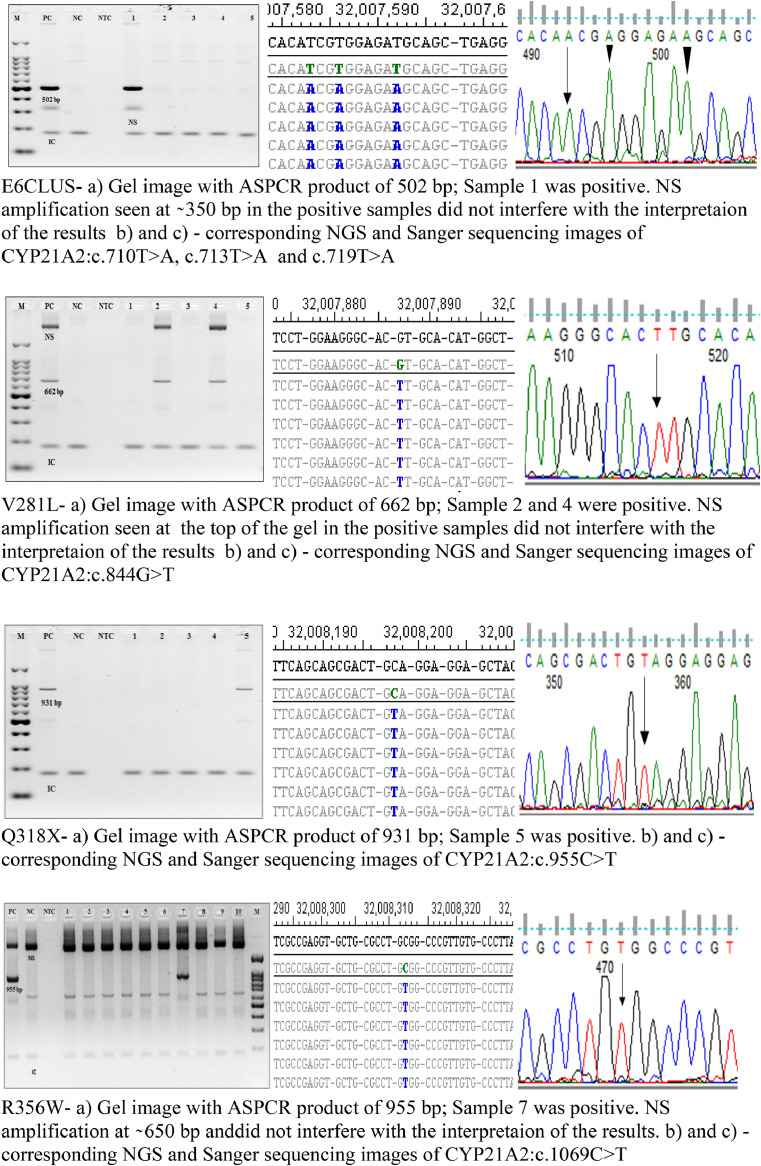
Gel images of ASPCR results, NGS alignments and chromatogram of Sanger sequencing results for the eight *CYP21A2* hotspot mutations screened. a) Agarose gel image (2%) showing ASPCR results for P30L, I2G, 8BPDEL, I172N, E6 CLUS, V281L, Q318X, and R356W mutation screening of different samples with appropriate Positive Control (PC), Negative Control (NC) and a No Template Control (NTC - To detect reagent contamination) run with mutant primers. IC indicates internal control at 180bp, 1 to n represent samples from different subjects, M indicates 100 bp marker.  NS (Non-specific) may indicate non-specific amplification from different combinations of the allele-specific and internal control primers. However, these non-specific products did not interfere with the identification of samples positive and negative for ASPCR. Utilizing these Allele-specific PCR the positive and negative control were compared with the test samples for genotyping.  b) and c) NGS results and chromatogram of Sanger validation of the eight hotspot mutations showing the same hotspot mutation corresponding to ASPCR. The chromosome coordinates of the NGS results indicate the alignment of the reads to the *CYP21A2* gene and not to the pseudogene *CYP21A1P*.

Following this, samples were screened for all the hotspot mutations with mutant primers, and the results were also validated with Sanger and NGS sequencing (Fig. 3).

#### *MLPA and ASPCR in identifying chimeric genes*

**Fig. 4:**
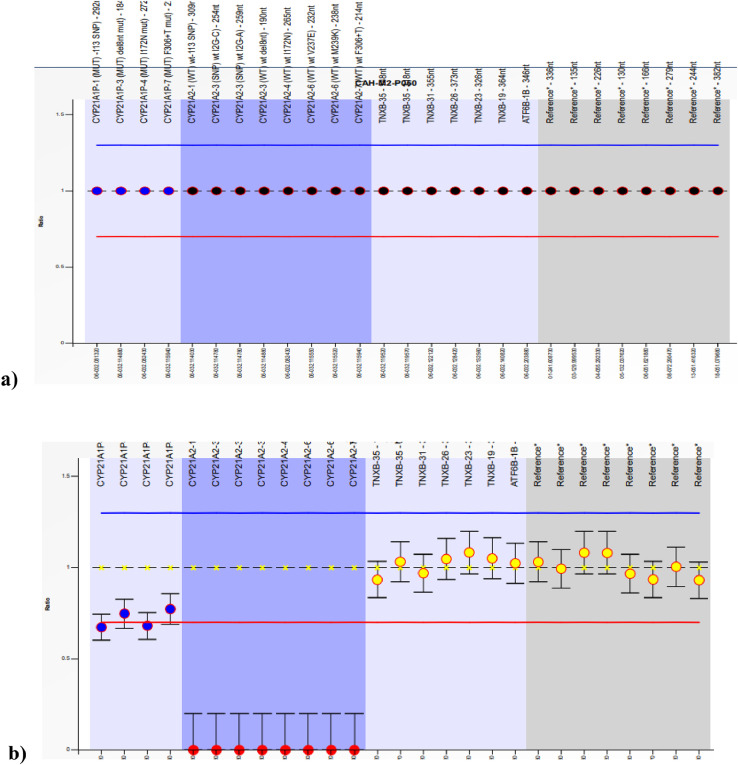

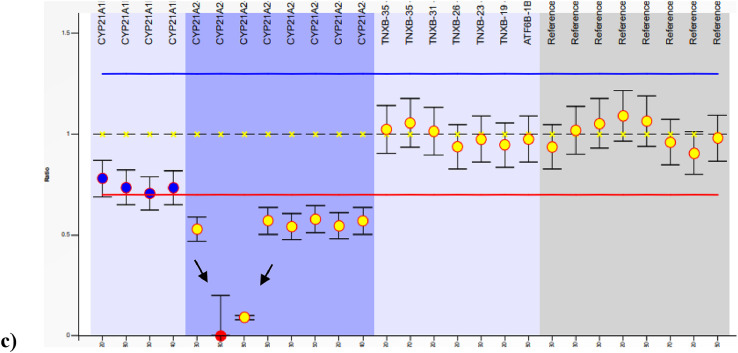
MLPA images of a) reference sample with copy number 1 for all the probes b) a sample positive for 30 kbp homozygous deletion with loss of eight probes in *CYP21A2* gene. This sample was homozygous positive for all the eight common mutations screened with ASPCR, indicating the formation of classic chimera CH8 [5]. c) A sample positive for heterozygous 30 kbp deletion with a copy number of 0.5 in several *CYP21A2* probes. The black arrowheads indicate the copy number of MLPA probes for Intron 2 splice site – each for wild type allele C and A to be zero with DQ of 0 and 0.09, respectively. With ASPCR, this subject was heterozygous for P30L, 8BPdel, I172N, E6CLUS and V281L and homozygous for I2G mutations. The parental screening revealed that the mother was a carrier for 30 kbp deletion and the father for the I2G splice variant. These results indicate that the subject is heterozygous for 30 kbp deletion with chimeric gene CH5 [5] on one allele and I2G splice mutation on the other allele.

Large 30 kbp deletion in 21 –hydroxylase deficiency results in the formation of chimeric genes involving the proximal end of *CYP21A1P* and the distal end of *CYP21A2* genes. MLPA (Multiplex Ligation-dependent Probe Amplification) is the most common technique employed in molecular analysis of large deletions and duplications in routine clinical practice. In this study, we utilized MLPA to validate large 30 kbp deletion suspected from the results of long-range PCR and restriction digestion using SALSA MLPA CAH Probemix P050 C1 from MRC-Holland [4]. Simultaneously allele-specific PCR was also carried out. Results of some of these samples are discussed below in Fig. 4.

The junction sites to classify classical and attenuated chimeras depend on the series of deleterious pseudogene mutations present in the extent of rearrangement. However, *CYP21A2* probes in the utilized MLPA assay span only till exon 7 out of 10 (probe: CYP21A2-7(WT) wt F306+T). But Q318X and R356W probes are also required to identify chimeras CH3 and CH8. Therefore, the ASPCR, including these mutations, is advantageous to identify the above chimeras.

### Multiplex PCR based target enrichment for NGS testing in CAH

**Fig. 5:**
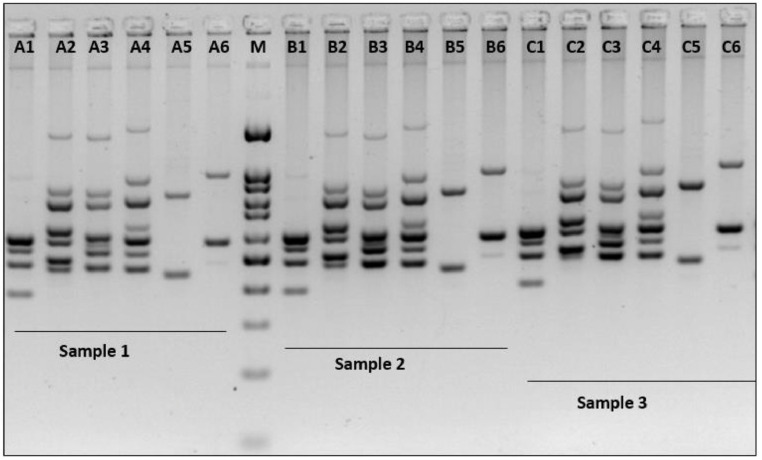
Agarose gel image (2%) of multiplex PCRs to amplify *CYP11B1, CYP17A1, CYP19A1 and POR* genes in 6 groups with 28 amplicons in three representative samples (A1-6, B1-6 and C1-6) respectively. 1-6 in each sample indicate amplicons amplified with the primer pool groups from 1 to 6 mentioned in Table 4

**Fig. 6:**
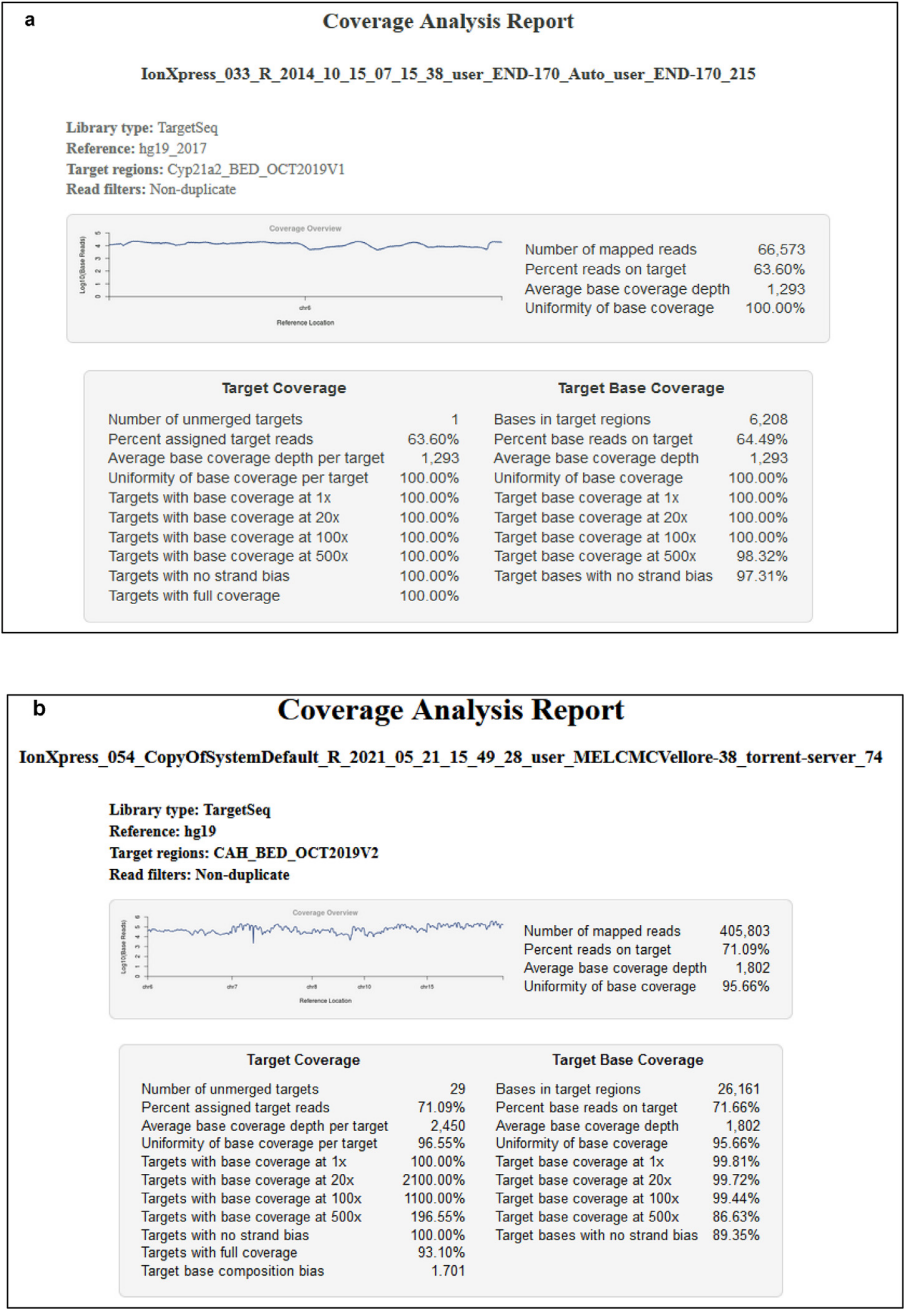
a. Coverage analysis report of a representative sample sequenced for *CYP21A2* gene with 100% of the target having a minimum coverage of 20X reads. **b**. Coverage analysis report of a representative sample sequenced for five genes CAH panel in 29 amplicons with 99.72% of the target having a minimum coverage of 20X reads and 99.44% of the target with 100X reads.

A multiplex PCR program was designed to comprehensively screen for *CYP21A2, CYP11B1, CYP17A1* and *POR* genes in CAH along with the *CYP19A1* gene that causes aromatase deficiency mimicking CAH. The coding and splice site regions of four genes - *CYP11B1, CYP17A1, POR* and *CYP19A1* were amplified in 28 amplicons in 6 groups. Primers for the *CYP11B1* gene were adapted from *white et al.* [6]. The primers were pooled into six groups based on the amplicon sizes (Table 3 and 4). The multiplex PCR was carried out using QIAGEN® Multiplex PCR kit. The PCR reaction mix and the conditions are described in table 5 and 6 respectively. The concentration of primers used was 10 pmol/µl. The PCR products were visualized on 2% agarose gel electrophoresis (Fig. 5.). Multiplex PCR products were pooled along with the long range PCR product of *CYP21A2* gene and sequencing with Ion Torrent PGM™ was performed following methods from published protocols [7]. Multiplex PCR coupled NGS sequencing achieved a uniform coverage across five genes with an average base coverage depth of 700X and with >99% of the target having 20X coverage (Table 7 and Fig. 6.).

**Table 3:** In house designed primer sequences for amplifying *CYP17A1, POR* and *CYP19A1* genes.

S.NO	PRIMER NAME	5' PRIMER SEQUENCE 3'
1	CYP17A1 EX1 F	TCCAAGCCTTGACTCCTGAG
2	CYP17A1 EX1 R	ACATGCACCTTCTCAGTCCA
3	CYP17A1 EX2-3 F	AAGGAAAGCAGGGACCAGAG
4	CYP17A1 EX2-3 R	AAAAGATGGGTCATTGCGGC
5	CYP17A1 EX4 F	CTCCTCCCTTGTTTAGAATTG
6	CYP17A1 EX4 R	CGCCCAGCCCTTAAGTCA
7	CYP17A1 EX5-6 F	CTGCCCAGACTTGCTCTACT
8	CYP17A1 EX5-6 R	AGTAGTTGATGGTTGACTGACTT
9	CYP17A1 EX7-8 F	AAACGCACACCCACATACAC
10	CYP17A1 EX7-8R	GAGCTCGAGTGTCCTGAGAA
11	POR EX1 F	CATTTCCTGCAGCCCCAG
12	POR EX1 R	TTTTCGCAGTGCTTCCTGTG
13	POR EX2 F	GGAATGTCCCCTCCCTGTG
14	POR EX2 R	CGGAGAGAAAATGGCAGTGG
15	POR EX3 F	GTGACCTTTGCCCTCCTTTG
16	POR EX3 R	GCAGGGATGGCAATGACC
17	POR EX4 F	GGCCTTCCCCATCTGGTG
18	POR EX4 R	GTCCACTGCCAGCCTCAA
19	POR EX5-6 F	GTCAACCAGATGAAGCCTCT
20	POR EX5-6 R	CTTCTAACCTTGCTGCGACC
21	POR EX7 F	TAGTCCAACCCCTCCCTCTC
22	POR EX7 R	TGCAGAGTAAGGTGGCTAAGT
23	POR EX8-9 F	GCCCTTGATGTAACCGGTGAGA
24	POR EX8-9 R	GCCTAAGCAGAAGCTCAACC
25	POR EX10-11 F	CCAGGGAGGCATCAGAGAG
26	POR EX10-11 R	GAGAATCTCACAAGCCAGCC
27	POR EX12-13 F	CTGCAGAACGGGACTTGG
28	POR EX12-13 R	AAGGGTGGTGCTGTGAGG
29	POR EX14-15 F	ACGAAGGTGGGCATGAGG
30	POR EX14-15 R	AAGTTGATGCAGGTGGAGGT
31	CYP19A1 EX 1F	CTTTGCCCTCCTTTCATCCAC
32	CYP19A1 EX 1R	TGCGACCAAATGTAGGGGAT
33	CYP19A1 EX 2F	GTCTTGCCTAAATGTCTGATCACA
34	CYP19A1 EX 2R	TTTCTCCCAAGTCCTCATTTGC
35	CYP19A1 EX 3F	ATGGAGAAGTGAAGAGCCTCAT
36	CYP19A1 EX 3R	TCAAGCAAAACCCAATTATTCTGTT
37	CYP19A1 EX 4F	ACAGAAGTGCTTATTCAACCCG
38	CYP19A1 EX 4R	CAAGGTCGTGAGCCAAGGTC
39	CYP19A1 EX 5F	CCTATCTCCTTCCGTTCATTCATT
40	CYP19A1 EX 5R	GCTGGCCCCTACTTTATGGAA
41	CYP19A1 EX 6F	TGGATGGCAAGGAGAACAAATC
42	CYP19A1 EX 6R	TCGACCCTTCTCTTCAACTCAA
43	CYP19A1 EX 7F	AGCTAACTCTGGCACCTTAACA
44	CYP19A1 EX 7R	GTGGGCTATTTGGATTGGGATT
45	CYP19A1 EX 8F	GTCCACAGTCAATCACAGAGAC
46	CYP19A1 EX 8R	AGAGGAGAGCGGAAAGGATTG
47	CYP19A1 EX 9F	GCATAACATATTTGGCCCTGGT
48	CYP19A1 EX 9R	GAAGGCTTGAGGATGAATACGG
49	CYP19A1 EX 10F	ACATAGAAAGGGCTTGAGTTCC
50	CYP19A1 EX 10R	CCTTGGGTTGAGGCAGTAGA
51	CYP11B1 EX1-2F*	TCGAAGGCAAGGCACCAG
52	CYP11B1 EX1-2R*	TGCTCCCAGCTCTCAGCT
53	CYP11B1 EX 3-5F*	AGAAAATCCCTCCCCCCTA
54	CYP11B1 EX3-5R*	GACACGTGGGCGCCGTGTGA
55	CYP11B1 EX 6-9F*	TGACCCTGCAGCTGTGTCT
56	CYP11B1 EX6-9R*	GAGACGTGATTAGTTGATGGC

⁎ Primers for the *CYP11B1* gene were adapted from *white et al.*[6].

**Table 4:** Grouping details of primers for multiplex PCR.

	Group No.	Product Size (bp)	volume (µl)
	GROUP 1		
1	POR EX7 F	387	10
	POR EX7 R		10
2	POR EX3 F	485	10
	POR EX3 R		10
3	POR EX1 F	545	10
	POR EX1 R		10
4	CYP19A1 EX 9F	589	10
	CYP19A1 EX 9R		10
		1X TE	70
		TOTAL	150
	**GROUP 2**		
1	CYP19A1 EX 4F	470	15
	CYP19A1 EX 4R		15
2	CYP19A1 EX 1F	506	10
	CYP19A1 EX 1R		10
3	POR EX4 F	581	10
	POR EX4 R		10
4	CYP19A1 EX 6F	627	10
	CYP19A1 EX 6R		10
5	CYP19A1 EX 10F	778	10
	CYP19A1 EX 10R		10
6	CYP11B1 EX1-2F	874	10
	CYP11B1 EX1-2R		10
7	CYP17A1 EX7-8 F	1448	10
	CYP17A1 EX7-8R		10
		1X TE	50
		TOTAL	200
	**GROUP 3**		
1	CYP19A1 EX 7F	468	10
	CYP19A1 EX 7R		10
2	CYP19A1 EX 3F	534	10
	CYP19A1 EX 3R		10
3	CYP19A1 EX 5F	600	10
	CYP19A1 EX 5R		10
4	POR EX10-11 F	768	10
	POR EX10-11 R		10
5	POR EX5-6 F	850	10
	POR EX5-6 R		10
6	CYP11B1 EX 3-5F	1409	10
	CYP11B1 EX3-5R		10
		1X TE	80
		TOTAL	200
	**GROUP 4**		
1	CYP19A1 EX 2F	471	10
	CYP19A1 EX 2R		10
2	POR EX2 F	527	10
	POR EX2 R		10
3	CYP19A1 EX 8F	585	10
	CYP19A1 EX 8R		10
4	POR EX12-13 F	649	20
	POR EX12-13 R		20
5	CYP17A1 EX2-3 F	795	10
	CYP17A1 EX2-3 R		10
6	CYP17A1 EX5-6 F	950	10
	CYP17A1 EX5-6 R		10
7	CYP11B1 EX 6-9F	1541	10
	CYP11B1 EX6-9R		10
		1X TE	40
		TOTAL	200
	**GROUP 5**		
1	CYP17A1 EX4 F	443	2
	CYP17A1 EX4 R		2
2	POR EX14-15 F	818	3
	POR EX14-15 R		3
		TOTAL	10
	**GROUP 6**		
1	POR EX8-9 F	566	1
	POR EX8-9 R		1
2	CYP17A1 EX1 F	972	1
	CYP17A1 EX1 R		1
		1X TE	6
		TOTAL	10

**Table 5:** Multiplex PCR reaction mix.

Contents	Volume
2x QIAGEN Multiplex PCR Master Mix Q solution DNA Primer pool Sterile water	7.5 µl 1.5 µl 1 µl 3 µl 2 µl
Total	15 µl

Primer concentration used: 10 pmol/µl.

**Table 6:** Multiplex PCR program.

Stage 1 × 1	Initial denaturation	95 °C	10 minutes
		98 °C	5 minutes
Stage 2 × 25	Denaturation	98 °C	30 seconds
	Annealing	60 °C	90 seconds
	Extension	72 °C	90 seconds
Stage 3 × 1	Final extension	72 °C	10 minutes

**Table 7:** Target coverage summary generated from Ion torrent coverage analysis plugin for CAH - 5 gene panel with 29 amplicons. The coverage of amplicons 17&18 and 19&20 are merged.

Amplicon No	Contig_start	Contig_end	Region ID	ave_base reads	fwd_base reads	rev_base reads	Cov 20x	Cov 100x	Cov 500x
1	104590181	104591627	chr10:104590181-104591627	517.914	415200	334222	1447	1420	824
2	104596499	104597469	chr10:104596499-104597469	1166.65	605352	527465	971	971	960
3	104592145	104593093	chr10:104592145-104593093	1713.268	952225	673666	949	949	949
4	104594438	104595231	chr10:104594438-104595231	2463.586	980654	975433	794	794	794
5	104593641	104594084	chr10:104593641-104594084	3555.056	879897	698548	442	442	442
6	51528876	51529408	chr15:51528876-51529408	2727.535	895187	558589	533	533	533
7	51510437	51511062	chr15:51510437-51511062	2763.912	657054	1073155	626	626	626
8	51502646	51503422	chr15:51502646-51503422	2783.369	1274280	888398	777	777	777
9	51514346	51514944	chr15:51514346-51514944	3165.851	837333	1059012	599	599	599
10	51506984	51507567	chr15:51506984-51507567	3259.106	964143	939175	584	584	584
11	51630548	51631052	chr15:51630548-51631052	3544.501	918710	871263	505	505	505
12	51504333	51504920	chr15:51504333-51504920	3768.374	1191552	1024252	588	588	588
13	51519816	51520284	chr15:51519816-51520284	4440.981	1244142	838678	469	469	469
14	51507754	51508220	chr15:51507754-51508220	4909.906	1122681	1170245	467	467	467
15	51534813	51535282	chr15:51534813-51535282	6990.323	1714667	1570785	470	470	470
16	32005398	32011605	chr6:32005398-32011605	808.098	2537725	2478950	6208	6208	4977
17 &18	75614821	75615956	chr7:75614821-75615468&chr7:75615407-75615956	591.27	285556	386127	1136	1136	872
19&20	75609528	75611028	chr7:75609528-75610224&chr7:75610180-75611028	1317.141	985186	991842	1431	1431	1339
21	75613940	75614706	chr7:75613940-75614706	1485.86	613624	526031	767	767	767
22	75583197	75583740	chr7:75583197-75583740	1953.465	603651	459034	544	544	544
23	75612700	75613264	chr7:75612700-75613264	2065.257	641878	524992	565	565	565
24	75601606	75602131	chr7:75601606-75602131	3238.409	886363	817040	526	526	526
25	75608573	75609056	chr7:75608573-75609056	3296.955	830463	765263	484	484	484
26	75611472	75611857	chr7:75611472-75611857	3574.839	689546	690342	386	386	386
27	143957567	143958974	chr8:143957567-143958974	391.872	291466	260290	1408	1360	233
28	143955781	143957320	chr8:143955781-143957320	1041.747	699797	904493	1540	1540	1509
29	143960421	143961293	chr8:143960421-143961293	1608.318	725207	678855	873	873	873

With the above comprehensive strategy, clinically significant variants were identified in *CYP21A2, CYP11B1* and *CYP19A1* genes in 97.2% of the study subjects (n=72) suspected for 21 hydroxylase and 11 beta hydroxylase deficiency. No disease-causing variants were identified in *CYP17A1* and *POR* genes. However, several polymorphisms were identified in the above two genes (table 8) indicating effective use of this CAH - NGS panel in clinical settings.

**Table 8:** List of polymorphisms identified in *CYP17A1* and *POR* genes through NGS strategy.

Subject ID	Gene	Ref Base	Called Base	Codon change	Protein change	Genotype	Effect	dbSNP ID	MAF in South Asians
C3	*CYP17A1*	G	A	c.138C>T	p.His46=	Homozygous	Synonymous	6162	0.476
		C	A|C	c.195G>T	p.Ser65=	Heterozygous	Synonymous	6163	0.359
	*POR*	G	A|G	c.1716G>A	p.Ser572=	Heterozygous	Synonymous	1057870	0.278
		A	G|A	c.387A>G	p.Pro129=	Heterozygous	Synonymous	1135612	0.206
		T	C	c.1455T>C	p.Ala485=	Homozygous	Synonymous	2228104	0.932
C30	*CYP17A1*	G	A	c.138C>T	p.His46=	Homozygous	Synonymous	6162	0.476
		C	A|C	c.195G>T	p.Ser65=	Heterozygous	Synonymous	6163	0.359
	*POR*	C	T|C	c.1508C>T	p.Ala503Val	Heterozygous	Non-synonymous	1057868	0.354
		T	C|T	c.1455T>C	p.Ala485=	Heterozygous	Synonymous	2228104	0.932
C31	*CYP17A1*	G	A|G	c.138C>T	p.His46=	Heterozygous	Synonymous	6162	0.476
	*POR*	C	T|C	c.1508C>T	p.Ala503Val	Heterozygous	Non-synonymous	1057868	0.354
		A	G|A	c.387A>G	p.Pro129=	Heterozygous	Synonymous	1135612	0.206
		T	C	c.1455T>C	p.Ala485=	Homozygous	Synonymous	2228104	0.932
C32	*CYP17A1*	G	A|G	c.138C>T	p.His46=	Heterozygous	Synonymous	6162	0.476
	*POR*	G	A|G	c.1716G>A	p.Ser572=	Heterozygous	Synonymous	1057870	0.278
		T	C	c.1455T>C	p.Ala485=	Homozygous	Synonymous	2228104	0.932
C46	*CYP17A1*	G	A	c.138C>T	p.His46=	Homozygous	Synonymous	6162	0.476
		C	A|C	c.195G>T	p.Ser65=	Heterozygous	Synonymous	6163	0.359
	*POR*	T	C	c.1455T>C	p.Ala485=	Homozygous	Synonymous	2228104	0.932
C47	*POR*	G	A	c.1716G>A	p.Ser572=	Homozygous	Synonymous	1057870	0.278
		T	C	c.1455T>C	p.Ala485=	Homozygous	Synonymous	2228104	0.932
C50	*CYP17A1*	G	A|G	c.138C>T	p.His46=	Heterozygous	Synonymous	6162	0.476
		C	A|C	c.195G>T	p.Ser65=	Heterozygous	Synonymous	6163	0.359
	*POR*	G	A|G	c.1716G>A	p.Ser572=	Heterozygous	Synonymous	1057870	0.278
		A	G|A	c.387A>G	p.Pro129=	Heterozygous	Synonymous	1135612	0.206
		T	C	c.1455T>C	p.Ala485=	Homozygous	Synonymous	2228104	0.932
C51	*POR*	G	A|G	c.1716G>A	p.Ser572=	Heterozygous	Synonymous	1057870	0.278
		A	G|A	c.387A>G	p.Pro129=	Heterozygous	Synonymous	1135612	0.206
		T	C	c.1455T>C	p.Ala485=	Homozygous	Synonymous	2228104	0.932
C52	*CYP17A1*	G	A	c.138C>T	p.His46=	Homozygous	Synonymous	6162	0.476
		C	A	c.195G>T	p.Ser65=	Homozygous	Synonymous	6163	0.359
	*POR*	C	T	c.1508C>T	p.Ala503Val	Homozygous	Non-synonymous	1057868	0.354
		T	C	c.1455T>C	p.Ala485=	Homozygous	Synonymous	2228104	0.932
C53	*CYP17A1*	G	A	c.138C>T	p.His46=	Heterozygous	Synonymous	6162	0.476
		C	A	c.195G>T	p.Ser65=	Heterozygous	Synonymous	6163	0.359
	*POR*	A	G	c.387A>G	p.Pro129=	Homozygous	Synonymous	1135612	0.206
		T	C	c.1455T>C	p.Ala485=	Homozygous	Synonymous	2228104	0.932
C54	*POR*	T	C	c.1455T>C	p.Ala485=	Homozygous	Synonymous	2228104	0.932
C55	*CYP17A1*	G	A	c.138C>T	p.His46=	Homozygous	Synonymous	6162	0.476
		C	A|C	c.195G>T	p.Ser65=	Heterozygous	Synonymous	6163	0.359
	*POR*	T	C	c.1455T>C	p.Ala485=	Homozygous	Synonymous	2228104	0.932
C56	*CYP17A1*	G	A	c.138C>T	p.His46=	Homozygous	Synonymous	6162	0.476
	*POR*	C	T	c.1508C>T	p.Ala503Val	Heterozygous	Non-synonymous	1057868	0.354
		T	C	c.1455T>C	p.Ala485=	Heterozygous	Synonymous	2228104	0.932

## Conclusion

The ASPCR assay was found to be highly specific and sensitive to detect all eight hotspot mutations in *CYP21A2* gene that were also identified by NGS and Sanger sequencing, validating its sensitivity and specificity. This assay is a simple cost-effective technique to genotype point mutations in *CYP21A2* gene and to identify junction sites in chimeric genes of *CYP21A2 - CYP21A1P* rearrangement that contributes to more than 90% of mutations in 21 – hydroxylase deficiency. Careful standardization enabled accurate and precise results that can provide a genetic diagnosis to a significant proportion of the CAH cohort in a clinical setting. The multiplex PCR assay enables a cost-effective step in NGS processing of CAH genes achieving uniform coverage matrices across the genes.

## Declaration of Competing Interest

The authors declare that they have no known competing financial interests or personal relationships that could have appeared to influence the work reported in this paper.
